# Retrosternal abscess after trigger point injections in a pregnant woman: a case report

**DOI:** 10.1186/1752-1947-5-403

**Published:** 2011-08-23

**Authors:** Faisal Usman, Abubakr Bajwa, Adil Shujaat, James Cury

**Affiliations:** 1 Department of Pulmonary & Critical Care, University of Florida College of Medicine, 655 West 8th Street, Jacksonville, Fl, USA 32209

## Abstract

**Introduction:**

Although retrosternal abscess is a well known complication of sternotomy and intravenous drug abuse, to date it has not been described as a consequence of trigger point injections. There are reported cases of serious complications as a result of this procedure including epidural abscess, necrotizing fasciitis, osteomyelitis and gas gangrene.

**Case presentation:**

A 37-year-old African-American woman, who was 20 weeks pregnant, presented to our emergency room with complaints of progressively worsening chest pain and shortness of breath over the course of the last two months. She was undergoing trigger point injections at multiple different sites including the sternoclavicular joint for chest pain and dystonia. Two years previously she had developed a left-sided pneumothorax as a result of this procedure, requiring chest tube placement and subsequent pleurodesis. Her vital signs in our emergency room were normal except for resting tachycardia, with a pulse of 100 beats per minute. A physical examination revealed swelling and tenderness of the sternal notch with tenderness to palpation over the left sternoclavicular joint. Laboratory data was significant for a white blood count of 13.3 × 10^9^/L with 82% granulocytes. A chest radiograph revealed left basilar scarring with blunting of the left costophrenic angle. A computed tomography angiogram showed a 4.7 cm abscess in the retrosternal region behind the manubrium with associated sclerosis and cortical irregularity of the manubrium and left clavicle.

**Conclusion:**

Trigger point injection is generally considered very safe. However, there are reported cases of serious complications as a result of this procedure. A computed tomography scan of the chest should strongly be considered in the evaluation of chest pain and shortness of breath of unclear etiology in patients with even a remote history of trigger point injections.

## Introduction

Retrosternal abscess is considered to be one of the most dreaded poststernotomy complications. There is a reported high incidence of retrosternal abscess in sternoclavicular joint infections regardless of any history of intravenous drug abuse, underlying illness or immunosuppression [1]. Retrosternal abscess may also develop secondary to mediastinitis, cardiopulmonary resuscitation or sternal bone marrow aspiration. *Staphylococcus *is the most commonly implicated organism in retrosternal abscess; other microorganisms include *Mycobacterium *species [2] and there is a reported case due to *Bartonella henselae*. To date, retrosternal abscess has not been described as a complication of trigger point injections.

### Case presentation

A 37-year-old African-American woman, who was 20 weeks pregnant, presented to our emergency room (ER) with complaints of chest pain and shortness of breath. These symptoms started two months previously and had progressively worsened. Approximately two years prior to this presentation she had had an evaluation for chest pain that included cardiac testing and a chest X-ray. Eventually she was diagnosed with dystonia and started on trigger point injections at multiple different sites including the left sternoclavicular joint. She developed a left-sided pneumothorax as the result of these injections and underwent video-assisted thoracoscopic pleurodesis for a refractory pneumothorax. She was symptom free for about 20 months. She subsequently had recurrence of her left-sided chest pain which was treated with episodic sternoclavicular joint trigger point injections. Our patient was not able to recall any of the medications used in the injection. Her chest pain progressively got worse and she developed shortness of breath one week prior to her current presentation. The chest pain was substernal, sharp, with 5/10 intensity, aggravated by breathing and had no relieving factors. She denied fever, chills, smoking or drug use. A review of systems was otherwise negative. Her vital signs in the ER were normal except for resting tachycardia, with a pulse of 100 beats per minutes. A physical examination revealed swelling and tenderness of the sternal notch with tenderness to palpation over her left sternoclavicular joint. The rest of her physical examination was normal.

Laboratory data was significant for a white blood cell count of 13.3 × 10^9^/L with 82% granulocytes. A chest radiograph revealed possible upper mediastinal widening and left basilar scarring with blunting of the left costophrenic angle. A computed tomography (CT) angiogram of her chest was performed, and revealed a 4.7 cm gas-containing abscess in the retrosternal region behind the manubrium, with associated sclerosis and cortical irregularity of the manubrium.

Our patient was started on broad spectrum antibiotics and then underwent surgical drainage and debridement of her superior mediastinum. All cultures, including those of the surgical specimen, were negative. She was treated for six weeks with intravenous vancomycin at home and recovered completely. She delivered a healthy full term baby.

## Discussion

Chronic pain is one of the most challenging medical problems in our society and the management of chronic pain has improved remarkably in recent years. Trigger points are focal hyperirritable areas in skeletal muscle which accompany many of the chronic musculoskeletal disorders. Trigger points may result in local or referred pain [3]. Acute or repetitive micro trauma to muscle fibers is the proposed mechanism of trigger points. Active trigger points are areas of tenderness to palpation that are accompanied by referred pain on compression, while latent trigger points cause referred pain but do not have pain at the site of the trigger point [4]. The distribution of trigger points can be quite random but the shoulder girdle, head, neck and lower back location are more commonly involved then the buttocks, knees or hips [5]. There are no specific tests or imaging studies required for the diagnosis of trigger points. The incidence of trigger points is typically higher in women. Trigger point injections are one of the most effective therapeutic approaches to treat this condition. The mechanisms related to inactivation of triggers include depolarization of nerve fibers from the extracellular shift of potassium due to mechanical disruption of muscle fibers, removal of metabolites due to vasodilatory effects from local anesthetics and the blockage of positive feedback for pain perception [6]. Different agents are used to inject trigger points and include saline, local anesthetics, steroids or combinations of these agents [7]. The procedure is generally considered to be very safe. The true incidence of complications is unknown, most likely due to underreporting.

Among the 276 claims associated with invasive procedures for chronic pain management in the American Society of Anesthesiologists Closed Claims Project, 17 cases involved trigger point injections [8]. Common complications occurring from trigger point injections are delineated in Appendix 1. The most common complication is infection including epidural abscess, necrotizing fasciitis, osteomyelitis and gas gangrene [6]. Non-infectious complications as a result of local mechanical injury or inflammatory response have also been described. These include spinal cord injury and peripheral nerve injuries, pneumothorax, air embolism, pain or swelling at the site of injection, and tendon and fascial ruptures [6]. Specific drug related reactions have also been described such as muscle weakness as a result of botulinum toxin injection [7]. There are no previously reported cases of retrosternal abscess formation as the result of trigger point injections.

## Conclusion

A CT scan of the chest should strongly be considered in the evaluation of chest pain and shortness of breath of unclear etiology in patients with even a remote history of trigger point injections.

## Abbreviations

CT: computed tomography; ER: emergency room.

## Consent

Written informed consent for publication of this case report and any accompanying images was obtained from the patient. A copy of the written consent is available for review by the Editor-in-Chief of this journal.

## Competing interests

The authors declare that they have no competing interests.

## Authors' contributions

FU made substantial contributions to the conception of this case report, acquired and interpreted data and drafted the manuscript. AB acquired and interpreted data and critically revised the manuscript for important intellectual. AS acquired and interpreted data and critically revised the manuscript for important intellectual. JC interpreted data and critically revised the manuscript for important intellectual content. All authors read and approved the final manuscript.

## Appendix 1

### Complications of trigger point injections

• Pneumothorax

• Epidural abscess

• Air embolism

• Intrathecal injection

• Skeletal muscle injury

• Osteomyelitis

• Necrotizing fasciitis

• Spinal cord injury and peripheral nerve injuries

• Pain or swelling at the site of injection

• Chemical meningism

• Granulomatous inflammation of the synovium

• Aseptic acute arthritis

• Embolia cutis medicamentosa

• Skeletal muscle toxicity

• Tendon and fascial ruptures

**Figure 1:**
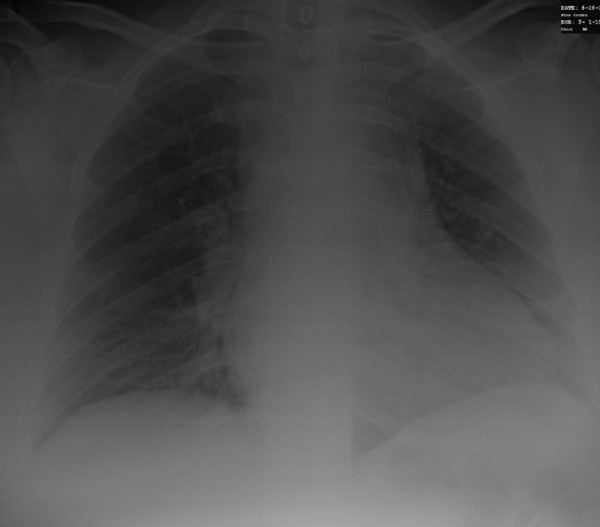
**Chest radiograph showing left basilar scarring with blunting of the left costophrenic angle**.

**Figure 2:**
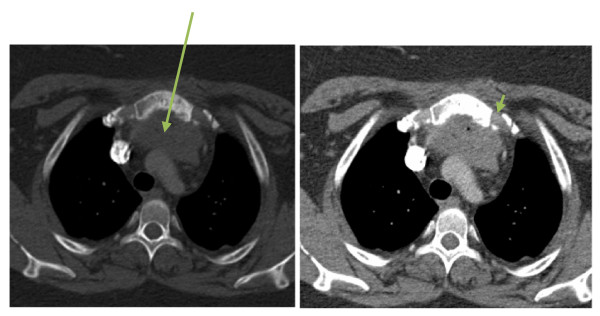
**CT scan of chest**. Mediastinal window: arrow shows retrosternal abscess behind the manubrium. Bone window: arrowhead showing cortical irregularity.
